# A multi-attack intrusion detection model based on Mosaic coded convolutional neural network and centralized encoding

**DOI:** 10.1371/journal.pone.0267910

**Published:** 2022-05-05

**Authors:** Rong Hu, Zhongying Wu, Yong Xu, Taotao Lai, Canyu Xia

**Affiliations:** 1 Fujian Provincial Key Laboratory of Big Data Mining and Application, Fujian University of Technology, Fuzhou, China; 2 Fujian Key Laboratory of Automotive Electronics and Electric Drive, Fujian University of Technology, Fuzhou, China; 3 Fujian Provincial Key Laboratory of Information Processing and Intelligent Control, Minjiang University, Fuzhou, China; 4 School of Chemistry and Environmental Engineering, Changchun University of Science and Technology, Changchun, China; Victoria University, AUSTRALIA

## Abstract

With the development of the Internet of Vehicles (IoV), attacks to the vehicle-mounted control area network (CAN) have seriously jeopardized the security of automobiles. As an important security measure, intrusion detection technologies have aroused great interest in researchers and many detection methods have also been proposed based on the vehicle’s CAN bus. However, many studies only considered one type of attack at a time but in real environments there may contain a variety of attack types simultaneously. In view of the deficiency in the current methods, this paper proposed a method to detect multi-intrusions at one time based on a Mosaic coded convolutional neural network (CNN) and a centralized coding method. A Mosaic-like data block was created to convert the one-dimensional CAN ID into a two-dimensional data grid for the CNN to effectively extract the data characteristics and maintain the time characteristics between the CAN IDs. Four types of attacks and all combinations of them were used to train and test our model. Finally, a centralized coding method was used to increase the discrimination capability of the model. Experimental results showed that this single model could successfully detect any combinations of the intrusion types with very high and stable performance.

## Introduction

The development of Internet technology brings convenience to people’s life, but also poses some threats to people’s privacy and security. Attackers can invade databases through the network to cause the leakage of user’s information [1]. As the Intelligent Transportation System (ITS) is becoming popular, each car has no longer been an isolated closed-loop system [2] and automobiles have also been developed towards the direction of heavily relying on information and intelligence technologies. More and more Electronic Control Units (ECUs) are integrated inside the automobile [3], which control the car’s internal subsystems, such as the Anti-lock Braking system, Advanced Driver-Assistance system, and In-Vehicle Infotainment system etc. [4]. The internal ECU communicates through the on-board network, and the Controller Area Network (CAN) is the most widely used on-board network protocol at present, which is also considered as the standard of modern automobile communication network [5]. The CAN network transmits messages that contain the control status of each ECU on the vehicle through the broadcast protocol. The vehicle-mounted network (VMN) interacts with the outside world through the vehicle-mounted gateway and at the same time allows the external network to affect the VMN environment [6], which rises the risk of attacks on the on-board network [7]. Therefore, vehicle on-board network security has become the research hotspot of the IoV technology. Intrusion Detection System (IDS), as an important technique of traditional computer network security protection, is also suitable for the use of vehicular network security. Compared with the traditional IDS, the vehicle network needs a more lightweight IDS due to the limitation of the ECU computing power and the communication bandwidth of CAN bus [8].

Although CAN bus can provide reliable communication, it also suffers from some security loopholes. Hackers can use these loopholes to launch attacks to cars, thus affecting the normal driving of them and even endangering the life safety of drivers [9]. In recent years, cyber attacks on cars have emerged in an endless stream, and there is an increasing trend every year [10]. In 2014, a study reported more than 20 information security models at the Black Hat Conference, and evaluated the ability of different models developed by different automobile manufacturers to resist malicious attacks [11]. In 2016, an attacker successfully interfered with a car’s powertrain and steering wheel by injecting an attack message through Jeep’s onboard diagnostic system interface [12]. Tencent’s Cohen Lab successfully hacked into Tesla electric vehicles remotely to control the ECU inside the car [13]. In 2017, Palanca *et al*. carried out a selective Denial of Service (DoS) attack on a car to interfere with the normal operation of it, which could occupy the resources of the CAN bus and thus paralyze it [14].

Due to the increasingly frequent network attacks, researchers have realized the importance of vehicle network security and put forward many countermeasures. Among the three security categories: authentication [15], firewall [16], and intrusion detection system [17], each has its own advantages and disadvantages. For example, the application of encryption and authentication technology is limited by the cost, bandwidth and storage resources of the vehicle-mounted system. The firewall strategy suffers from the long updating and upgrading cycle of the automotive electronic system and cannot be deployed on a large scale in the short term [18]. Nevertheless, the intrusion detection method can meet the real-time requirement of network protection and give an early warning to prevent further damage to the vehicles caused by attacks. The lightweight vehicular intrusion detection system can also avoid the impact to the computing power and bandwidth of vehicle equipment [19]. Therefore, this paper focuses on the study of an intrusion detection system of VMN for real-time detection of attacks to ensure the safety of vehicles.

According to the property of periodical transmission of CAN ID in vehicle-mounted CAN, researchers have proposed different detection methods. Miller *et al*. proposed an intrusion detection method based on frequency distribution [20]. When there is an abnormal signal in the VMN, the transmission frequency balance of CAN ID is broken, so as to judge the existence of attack behavior in the system. Muter *et al*. proposed an entropy-based intrusion detection method. By setting an entropy value on the CAN bus as a judgment threshold, the real-time detection and calculation of entropy on the bus were compared with the threshold value to judge whether there was an attack to the VMN [21]. Larson *et al*. proposed a canonical attack detection method to show whether the message complied with the ECU code of conduct so as to judge whether there was an attack in the network [22]. Lee *et al*. analyzed the response time and compensation rate of remote frames in CAN protocol to determine whether there was malicious attack in the system [23]. The method of anomaly detection for vehicular CAN network based on the periodicity of CAN ID transmission has limitations. Only when the attack is to inject a large number of specific CAN IDs into the network and violates the CAN protocol specification, can it be better detected [24].

In recent years, with the development of artificial intelligence, a large number of intrusion detection methods based on machine learning approaches have been proposed [25]. Alshammari *et al*. proposed an intrusion detection method based on K-nearest neighbors and support vector machine models by extracting the data field of the CAN message, which may cause privacy leakage and increase the computational cost [26]. Kang *et al*. constructed a classifier through the structure of a deep belief network, but they did not verify the validity of the model through real data sets [27]. Taylor *et al*. proposed a detection model based on long short-term memory. Although this model can effectively detect the attack behavior, it will increase the computational complexity and storage cost because it has too many parameters [28].

Convolutional Neural Networks (CNN), as one of the most popular models in the field of deep learning, has also been used in the vehicular network intrusion detection. Seo *et al*. proposed a classification model of intrusion detection by combining the Generative Adversarial Networks and Deep Neural Networks. The training and testing process of this method was complex and threshold values needed to be set to improve the classification capability [29]. Song *et al*. proposed an intrusion detection method based on an Inception-ResNet CNN model by encoding a CAN ID into a 29-bit binary vector, and then combining 29 such CAN IDs consecutively to form a 29-by-29 two-dimensional data [30]. However, all the methods proposed by now were only able to detect one kind of intrusion at a time. When there are multiple attacks, multiple models should be created to detect each of them but not a combination of them. Therefore, we aim to create a model that can be used to detect any combination of intrusion attacks by using a single CNN model combined with the Mosaic coded CAN IDs and centralized coding method. Experimental results showed that our model was not only able to detect any combination of multiple intrusion attacks but also performed better than the existing ones with much higher stabilization.

The main contribution of this paper is as follows:

1. Multiple attacks were considered in our model so that it was able to deal with the real attack environment in which hackers may attack vehicles with multiple attack types at the same time or with one attack type at one time and another type at another time in order to achieve a maximum attack effect. In this paper, four attack types (DoS, Fuzzy, Gear and RPM attacks) were used to train our network and all possible combinations of them were used to test our model. Although with the increase of attack types, the types and quantity of CAN IDs were also increased, which increased the complexity of the model classification, the model was still able to identify multiple attacks with very high and stable accuracy.
2. Mosaic coded CAN IDs were used to improve performance of the CNN model. As CNN is good at handling two-dimensional grid data such as an image, we first converted the one-dimensional CAN ID into a two-dimensional data grid, and then joined such grids together to form a Mosaic-like data block as a two-dimensional image for the CNN to recognize. This makes the CNN effectively extract the data characteristics and maintain the time characteristics between the CAN IDs.
3. Centralized coding method was used to increase the discrimination capability of the model. Since the identifier (CAN ID) is a binary number represented by digits ’0’ and ’1’ and most of them are 0s, 0 would not response to any change in weight and when the difference between two IDs is only one digit, the degree of discrimination between them is not high. However, when the centralized coding method was adopted, all 0s were eliminated and the degree of differentiation between different IDs would be much improved even if they only have one digit difference.

## Background knowledge

### Automotive CAN bus network

CAN bus network was proposed by Bosch Company in 1983 to facilitate the communication among ECUs inside the vehicle. It is now widely used in vehicle network systems because it has the characteristics of self-diagnosis and avoiding electronic interference. Although CAN bus can provide reliable communication functions, with the development of the IoV, its security issues have gradually made obvious [31]. However, although other VMNs such as Local Interconnect Network and FlexRay have also been proposed, CAN bus network is still the mainstream VMN protocol used by now.

CAN network divides ECUs into multiple subnetwork systems according to their functions, and each subnetwork is communicated with each other and all of them connected together through the vehicular gateway to form a bus topology structure. It is generally composed of a high-speed bus with a transmission rate of 500kbps and a low-speed bus with a rate of 100kbps. Each ECU in CAN bus includes three parts: microcontroller, CAN controller and CAN transceiver. It uses two twisted pair wires for electrical signal transmission which are divided into the high data line (CAN_H) and low data line (CAN_L), and the terminals are connected to a resistor of 120 Ω. The voltage state of the CAN bus is determined by the potential difference between CAN_H and CAN_L of the two twisted pair wires and the logic 0 is defined as the dominant bit and logic 1 as the implicit bit.

**Fig 1:**
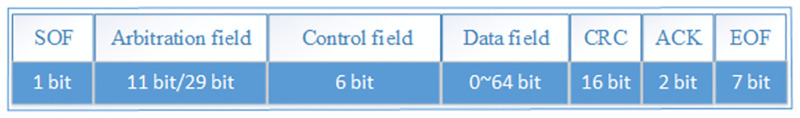
Structure of the CAN message.

CAN bus data frame packet is mainly composed of CAN identifier, control domain, data domain, cyclic redundancy check, ACK, etc. as is shown in Fig 1. The packet’s format is divided into CAN 2.0A standard frame format and CAN 2.0B expansion frame format. The main difference between these two formats lies in the number of CAN identifiers. CAN ID of the former contains 11 bits of binary number, while the latter contains 29 bits of binary number. CAN bus sends messages via a broadcast protocol. When one ECU sends a message, other ECU nodes can receive the message. If more than one ECU on the bus requests to send data at the same time, the priority arbitration mechanism in the CAN bus judges the priority of the data according to the CAN ID. The dominant bit has higher priority than the implicit bit, and when one node wins the arbitration, the other node is put into to the waiting state. In addition, the transmission of messages on the vehicle CAN bus is periodic.

### Security issues of CAN bus

Previously, the security issues of CAN bus did not get enough attention, so there are some security loopholes in CAN bus protocol, such as the data have no encryption and no authentication and the authenticity of messages cannot be accurately determined. Some hackers take advantage of these security vulnerabilities to launch malicious attacks on cars via remote or local access. Common attack modes on the CAN bus include: (1) DoS attack. Attackers inject a large number of data packets with high priority into CAN bus in a short time. For example, the highest priority ID 0x000 is used to occupy the CAN bus to paralyze it. (2) Fuzzy attack. Attackers inject randomly generated packet data into CAN bus, and the nodes in the network receive a large number of intrusion messages, leading to abnormal functionality of the vehicle. (3) Spoofing attack. The attacker takes control of the message transmission of nodes so that they can no longer send useful messages to the network. The attacker can also use simulated nodes to respond to the network so as to cheat the node to realize that it seems not being controlled by the attacker.

### Convolutional neural network

CNN is the most widely used deep learning model in the field of image recognition and classification, which is mainly composed of convolutional layer, pooling layer and full connection layer. It has the properties of weight sharing, dimensionality reduction and translation invariance etc.

**Fig 2:**
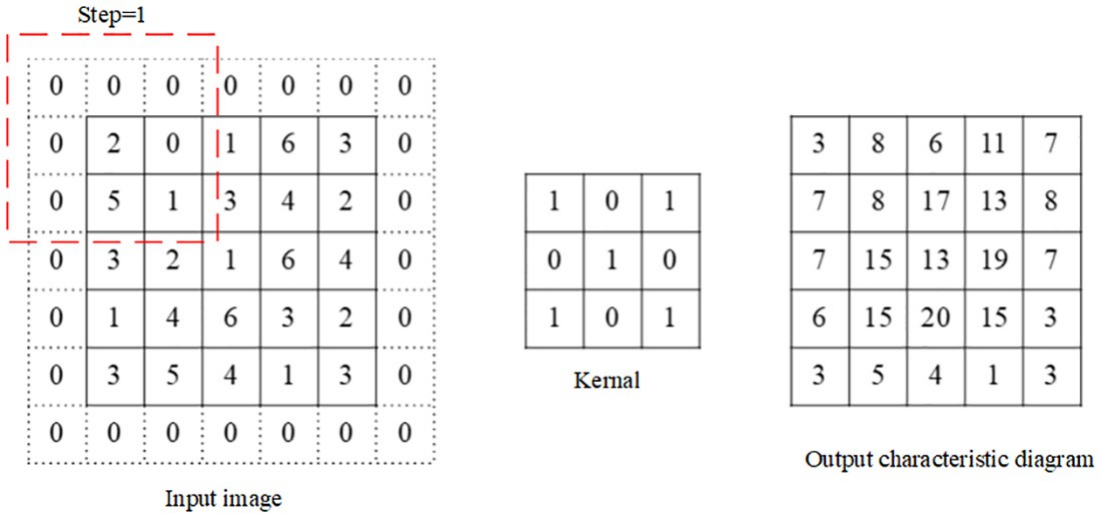
Schematic diagram of two-dimensional image convolution operation with paddings.

**Fig 3:**
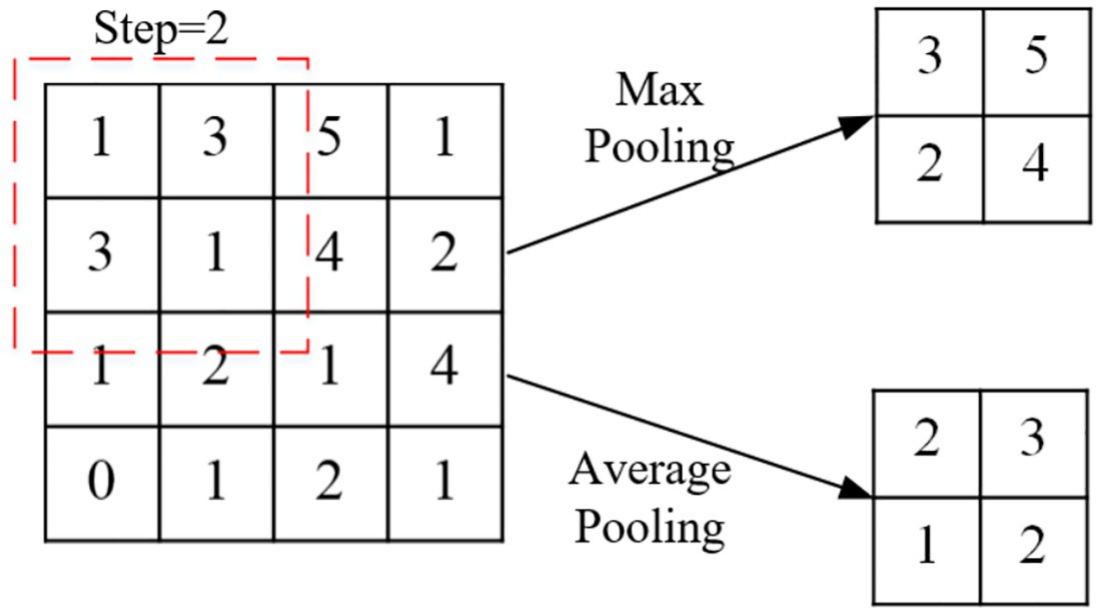
Maximum and average pooling operations during pooling.

The operation of convolution is shown in Fig 2, in which the image size is 5×5, convolution kernel size is 3×3, step size is 1 and the padding size with zero filling is 1. The operation of pooling is shown in Fig 3, in which the size of the pooling kernel is 2×2 and the step size is also 2. The upright graph shows the maximum pooling and downright graph the average pooling, both of which reduced the dimensionality of the image by half after the pooling operation.

The full connection layer is similar to a feedforward neural network for classification. Its calculation is shown as follows:

$$y\left(x\right)=f(W*X+B)$$ (1)

where *W* is the weight matrix, *X* is the input vector, *B* is the bias vector, and *f* is the activation function.

In addition, instead of arranging the CAN ID sequentially to form a data grid [30], which cannot make full use of the characteristics of CNN to recognize two-dimensional image features and is unable to maintain the temporal characteristics of CAN IDs, an improved coding method was proposed in this paper to enable CNN to extract data features more effectively to further improve the classification accuracy, which will be explained in detail in the next section.

## Method

### Two-dimensional Mosaic pattern based encoding

**Fig 4:**
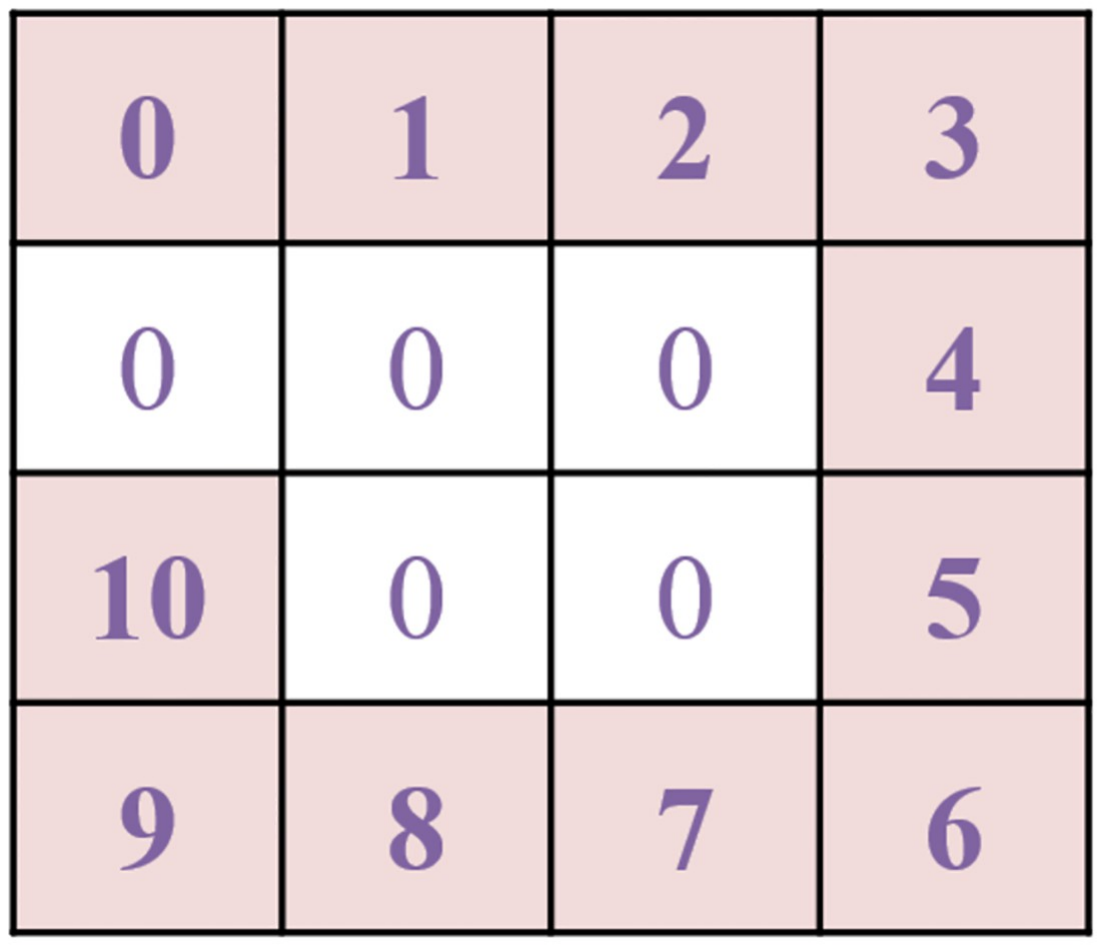
A 4×4 data grid for a 11-bit CAN ID.

**Fig 5:**
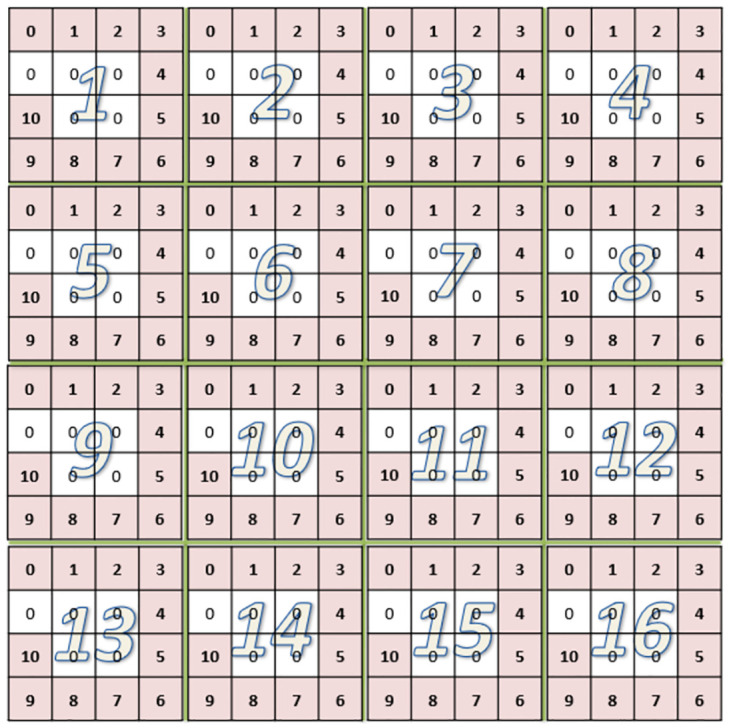
16 4×4 data grids arranged sequentially one after another to form a Mosaic data structure as the network input to keep the time feature of the original data.

In our coding method, each one-dimensional binary CAN ID was first converted to a 2D data grid then a number of such data grids were put together to form a two-dimensional Mosaic pattern. A Mosaic grid is defined in this paper as a data grid formed by CAN IDs by coding each of them as a square grid and then connecting them one after another to form a larger square pattern. For a 11-bit CAN ID, a 4×4 data grid was formed in which the 11-bit binary numbers were first filled around the 4 edges of the grid and then the remaining 5 elements were filled by zero. The result is shown in Fig 4, where the numbers in the grid represent the bits in binary. The reason of filling the data in this way is that the patterns surrounding the grid could more easily be recognized by the CNN. The two-dimensional data grid was then further arranged to form a larger two-dimensional Mosaic structure in the form of 8×8 data patterns, a reduced version of which is shown in Fig 5. As CNN is only good at handling image-like two-dimensional grid data, by coding CAN IDs in such Mosaic structure, not only can the temporal relationship among sequential data features be kept by arranging one data grid after another from the continuous data series, but the recognition ability of the CNN can also be improved. 64 such 4×4 data grids form a 32×32 element Mosaic pattern. The converted Mosaic data patterns will be used as the training and test sets of the CNN to preserve the time characteristics of the original data.

**Fig 6:**
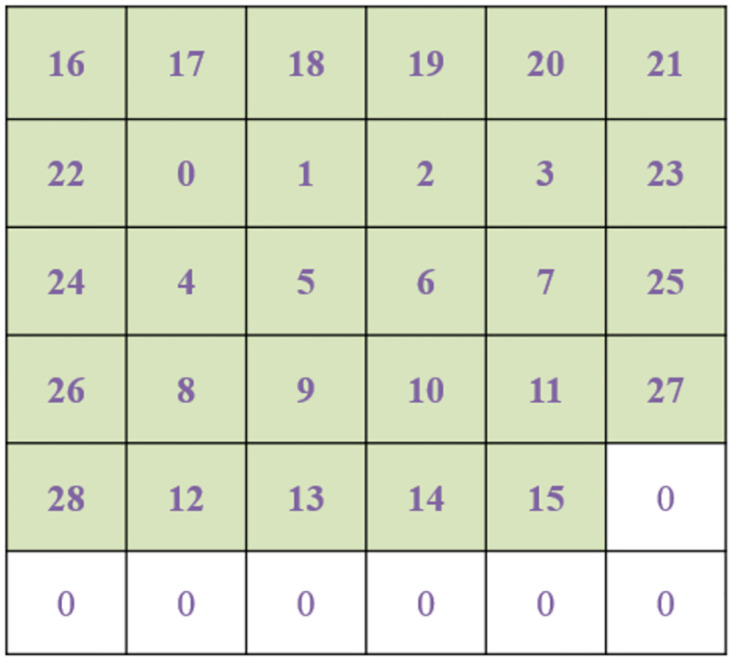
A 6×6 data grid for a 29-bit CAN ID.

If the 29-bit CAN ID data was used, it would be converted to a 6×6 data grid. Currently, as only the first 11 bits of data in the total 29 bits are nonzero, they will be put around the center of the 6×6 grid as is shown in Fig 6 where the numbers in the grid also represent the bits in binary. Since there are 36 elements in the 6×6 grid, 29 of them were filled by the CAN ID and the remaining 7 elements were filled with zero. In this way, it is also easier for the first 11 nonzero CAN ID patterns to be recognized by the CNN. Similarly, after the data were gridded, they were put together to form a Mosaic structure as it was shown in Fig 5 to maintain the time characteristics between each piece of data. In this paper, 8×8 data grids were used to form a 48×48 Mosaic structure, which served as the input and test sets of the network.

### Centralizing the data in Mosaic coding

Since CAN ID is a binary data, the value of each bit B in the CAN ID can only be 0 or 1 and most of the ID bits are 0. When a bit of data B in the CAN ID is 0, it will have no effect on the convolution operation. Therefore, in order to increase the discriminability during the convolution operation, the data were centralized to eliminate all 0s so that each bit could take part in the convolution operation. The data centralization method is given in the following formula:

C=B-(1-avge) (2)

where C represents the ID bit after centralization, B is the ID bit before centralization (0 or 1), and *avge* represents the average of either 11 or 29 bits of CAN ID binary data.

**Fig 7:**
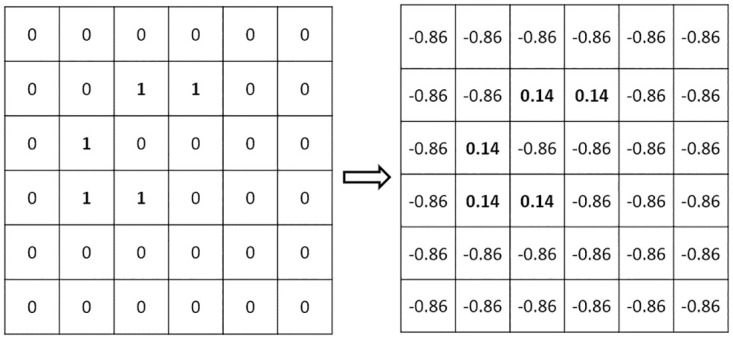
Data Centralization results.

When B = 0, the centralized result is a negative number and when B = 1, it is positive. In this way, each binary data in the CAN ID becomes a nonzero number (except when CAN ID = 0x0000). For example, when CAN ID = 0x0316, the centralized result is shown in Fig 7. We can see from this Figure that all 0s were eliminated so that each bit in the new CAN ID would be able to function in the convolution operation. Furthermore, when the CAN ID is changed in only one bit, the average value of it would be different and after centralization each CAN ID bit would have different positive or negative values. In this way, the new CAN ID would not only have different appearance but also have different digital values to achieve the purpose of improving the distinguishability between different CAN IDs.

After the data grids have been centralized, they would be used to form the Mosaic structures in the way described in the previous subsection as the training and test sets.

### The CNN model

In order to test the superiority of our coding method, the CNN model used in this paper only consists of an input layer, a convolution layer, a pooling layer, a full connection layer and an output layer. The input is the two-dimensional Mosaic grid data described in the previous two subsections.

20 convolution kernels were used in the convolution layer. The output size of the convolution layer can be worked out using Eq (3), where O is the output size, W is the data size, K is the kernel size, P is the padding method, and S is the step size. The padding P was used to ensure that the data size after convolution is the same as that of the input.


$$O=\frac{W-K+2P}{S}+1$$ (3)

When there are multiple convolution layers, the input of a convolution is the output of the last layer and the output of it serves as the input of next layer. The output of each convolution layer can be worked out by Eq (4), where *a*^*i*^ represents the output of a convolution layer, *σ* is the activation function, *a*^*i*−1^ is the output of the last layer, *W*^*i*^ is the connection weight, and *b*^*i*^ is the bias of the layer. In this paper, the bias *b*^*i*^ was set with an initial value of 0.1.


$$a^{i}=\sigma \left(z\right)=\sigma (a^{i-1}*W^{i}+b^{i})$$ (4)

The dimension of pooling window F was 2×2 and the average pooling was used for each non-overlapped 2×2 region. Therefore, the dimension of output data after pooling was reduced to half of the original one. There is the full connection layer after the pooling layer. 128 neurons were used in this layer which were connected to the neurons of the flattened pooling layer. The bias value b in the 128 neurons was also set with an initial value of 0.1, and tanh was used as the activation function. In order to prevent from overfitting in the process of model training, a dropout layer was used which was realized by making the activation function in some neurons fail with a certain probability. In the training process, the failure probability in the dropout layer was set as 0.5, but during the test, no dropout was used.

Finally, it is the output layer. Since there were only two types of output, i.e., normal or attack, two neurons were used in the output layer, which were fully connected to the 128 neurons in the previous layer. The softmax classifier shown in Eq (5) was used at this level to classify these two categories, as it is more suitable for multiple classification tasks than other activation functions.

$$P_{i}=\frac{e^{o_{i}}}{\sum_{i=1}^{n}e^{o_{i}}}$$ (5)

where *n* is the number of classes (in this paper *n* = 2), *o*_*i*_ is the output *i* of the classification and *P*_*i*_ is the probability of output *i*.

**Fig 8:**
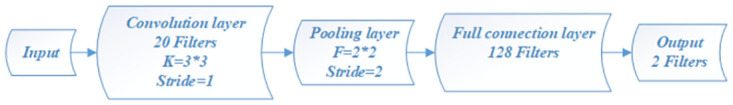
CNN model structure.

Finally, one-hot encoding was used as the output, i.e., when one of the two outputs is greater than the other, this one will be set as true or 1, and the other neuron is false or 0. This is equivalent to setting the threshold as 0.5. However, this would lead to a low classification reliability as even when the output is only slightly greater than 0.5 (say, 0.51), it will be classified to certain category. Therefore, higher thresholds, namely 0.5, 0.6, 0.7, 0.8 and 0.9, were set in this paper to increase the reliability of the classification by the model. When the probability of one of the two final output neurons is greater than the threshold (another output must be lower than one minors the threshold), the category corresponding to that output will be taken as the discriminant result. Otherwise, it will be classified as an unrecognized sample. In this way, a higher standard of classification could be set by ruling out some unrecognized samples. The structure of the CNN is shown in Fig 8.

In the optimization of the CNN, the cross entropy was selected as the loss function and the adaptive moment estimation optimization method was used to minimize the function. By constantly adjusting the learning rate, the optimal learning rate of the optimizer was found to be 1e-4.

The model was trained and tested using the VMN attack data collected by Hacking and Countermeasure Research Lab in South Korea [32]. This data includes four data sets: DOS, Fuzzy, Spoofing Gear and Spoofing RPM attacks. The data recorded CAN traffic through the onboard diagnostic (OBD-II) port of a real vehicle when the attack message was injected. During the process of data collection, the engine of the test vehicle remained on. Each data set had a total of 30–40 minutes of CAN traffic and contained 300 intrusions of the injected message, each continued for 3–5 seconds.

## Experimental results and discussions

Our proposed CNN model was tested in a series of experiments. In order to evaluate the effectiveness of the proposed model, a combination of the four data sets mentioned above was used as the training and test sets to meet the requirements of multi-type attack detection. The training set contained a randomly selected 75% of the data and the remaining 25% was used as the test set. Although the model was trained using the combination of all four sets of data, in the test phase, all the possible combinations of the data sets were used for test. Therefore, the test sets contained $C_{4}^{1}=4$ combinations of one kind of attack, $C_{4}^{2}=6$ combinations of two kinds of attacks, $C_{4}^{3}=4$ combinations of three kinds of attacks, and $C_{4}^{4}=1$ combination of four kinds of attacks, with a total of 15 different attack types. With this type of training and test, our model would be able to detect any intrusion combinations in the future, which is different from the current ones which were only able to detect a single type of attack after being trained.

### Experimental results

The detection of the intrusion attack is actually a dichotomous task with only two classification categories, i.e., positive (attack message) and negative (normal message). Therefore, the dichotomous confusion matrix and its related evaluation indexes were adopted to evaluate the performance of the model. Since the Mosaic pattern used as the training and test sets contains more than one CAN ID, if any of these CAN IDs contains at least one attack sample, it will be marked as an attack (positive P). Otherwise, it will be marked as normal (negative N). Under this circumstance, the specific meanings of True/False positives and True/False negatives are described as follows.

1. True positives (TP): The number of positive samples correctly classified by the model, i.e., the input data containing at least one attack sample is correctly identified as attack.
2. False positives (FP): The number of negative samples that are wrongly classified as positive by the model, i.e., the input data not containing any attack sample is misidentified as attack.
3. False negatives (FN): The number of positive samples that are wrongly classified as negative by the model, i.e., the input data containing at least one attack sample is misidentified as normal.
4. True Negatives (TN): The number of negative samples correctly classified by the model, i.e., the input data not containing any attack sample is correctly identified as normal.

Apart from the confusion matrix, there were some other indexes based on it were adopted to evaluate the results, which are described as follows:

1. Unrecognizable rate (*UR*): the number of unrecognized samples divided by the total number of samples processed in a specific coding scenario, which can effectively reflect the reliability of the model. When the threshold was set greater than 0.5, some of the samples would be unrecognized that could be either a normal input or an attack. It can be worked out by the following equation (where U is the number of unrecognized samples):
$$UR=\frac{U}{Sum}$$ (6)
2. Accuracy (*Acc*): the number of correctly classified samples over the total test samples. Generally, the higher the accuracy, the better the classifier. It is defined as:
$$Acc=\frac{TP+TN}{Sum}$$ (7)
3. Recall *(Rec*): a measure of coverage, indicating how many positive samples are correctly classified as positive. It is defined as:
$$Rec=\frac{TP}{TP+FN}$$ (8)
4. False Negative Rate (*FNR*): the percentage of mis-classified positive samples, which is defined as:
$$FNR=\frac{FN}{TP+FN}$$ (9)
5. Precision (*Prec*): a measure of accuracy, representing the percentage of correctly classified positive samples among all classified positive samples. It is defined as:
$$Prec=\frac{TP}{TP+FP}$$ (10)
6. Comprehensive classification rate (*F1_score*): the harmonic mean (a weighted average) of model accuracy rate and recall rate, reflecting the overall accuracy of the model. It is defined as:
$$F1\_score=\frac{2*Prec*Rec}{Prec+Rec}$$ (11)

Using the CNN model, we tested both 11-bit and 29-bit CAN IDs. When the 11-bit CAN ID was used, we first encoded the CAN IDs using the method described in [30] by directly putting one ID after another to form a data grid of 16×16 as the input of the model. Then, the Mosaic coding method was used to convert the 11-bit CAN ID to a 4×4 data grid and 8×8 of such data grids were put together to form a 32×32 Mosaic pattern as the input data of the model. In the experiments, we conducted 10 independent tests and took the mean of them. The mean and the corresponding standard deviation results of different coding methods are shown in Tables 1 and 2. Experimental results with the direct and the Mosaic coding methods and the centralized data processing method for the 11-bit CAN ID with all four attack data under different thresholds (0.5, 0.6, 0.7, 0.8 and 0.9) are shown in Table 1. According to the experimental results in Table 1, under different threshold conditions, we can see that the Mosaic coding methods not only performed better in all evaluation indexes than that of the 16×16 direct sequential coding method, but also ran more stable by giving a much lower standard deviation. We can also see that the centralized coding method performed the best among all the evaluation indexes compared with those with no centralization processing and also gave the most stable results.

**Table 1:** The 11-bit CAN ID test results for all four attacks.

***Threshold = 0*.*5***	*Prec*	*Rec*	*F1_score*	*Acc*	*FNR*
*16×16 sequential*	*0*.*9912*±*0*.*0013*	*0*.*9551*±*0*.*0053*	*0*.*9728*±*0*.*0032*	*0*.*9797*±*0*.*0023*	*0*.*0449*±*0*.*0053*
*32×32 Mosaic*	*0*.*9975*±*0*.*0005*	*0*.*9900*±*0*.*0013*	*0*.*9937*±*0*.*0008*	*0*.*9952*±*0*.*0006*	*0*.*0100*±*0*.*0013*
***32×32 Mosaic centralized***	***0*.*9983*±*0*.*0003***	***0*.*9923*±*0*.*0004***	***0*.*9953*±*0*.*0001***	***0*.*9964*±*0*.*0001***	***0*.*0077*±*0*.*0004***
***Threshold = 0*.*6***	*Prec*	*Rec*	*F1_score*	*Acc*	*FNR*
*16×16 sequential*	*0*.*9935*±*0*.*0009*	*0*.*9597*±*0*.*0048*	*0*.*9763*±*0*.*0027*	*0*.*9756*±*0*.*0028*	*0*.*0403*±*0*.*0048*
*32×32 Mosaic*	*0*.*9980*±*0*.*0004*	*0*.*9909*±*0*.*0011*	*0*.*9944*±*0*.*0007*	*0*.*9945*±*0*.*0007*	*0*.*0091*±*0*.*0011*
***32×32 Mosaic centralized***	***0*.*9987*±*0*.*0003***	***0*.*9929*±*0*.*0004***	***0*.*9958*±*0*.*0001***	***0*.*9959*±*0*.*0001***	***0*.*0071*±*0*.*0004***
***Threshold = 0*.*7***	*Prec*	*Rec*	*F1_score*	*Acc*	*FNR*
*16×16 sequential*	*0*.*9952*±*0*.*0007*	*0*.*9649*±*0*.*0042*	*0*.*9798*±*0*.*0023*	*0*.*9705*±*0*.*0037*	*0*.*0351*±*0*.*0042*
*32×32 Mosaic*	*0*.*9985*±*0*.*0003*	*0*.*9919*±*0*.*0010*	*0*.*9952*±*0*.*0006*	*0*.*9936*±*0*.*0009*	*0*.*0081*±*0*.*0010*
***32×32 Mosaic centralized***	***0*.*9990*±*0*.*0002***	***0*.*9935*±*0*.*0003***	***0*.*9962*±*0*.*0001***	***0*.*9954*±*0*.*0002***	***0*.*0065*±*0*.*0003***
***Threshold = 0*.*8***	*Prec*	*Rec*	*F1_score*	*Acc*	*FNR*
*16×16 sequential*	*0*.*9966*±*0*.*0006*	*0*.*9704*±*0*.*0036*	*0*.*9833*±*0*.*002*	*0*.*9618*±*0*.*0056*	*0*.*0296*±*0*.*0036*
*32×32 Mosaic*	*0*.*9989*±*0*.*0001*	*0*.*9931*±*0*.*0008*	*0*.*9960*±*0*.*0004*	*0*.*9921*±*0*.*0012*	*0*.*0069*±*0*.*0008*
***32×32 Mosaic centralized***	***0*.*9992*±*0*.*0001***	***0*.*9942*±*0*.*0003***	***0*.*9967*±*0*.*0001***	***0*.*9947*±*0*.*0002***	***0*.*0058*±*0*.*0003***
***Threshold = 0*.*9***	*Prec*	*Rec*	*F1_score*	*Acc*	*FNR*
*16×16 sequential*	*0*.*9978*±*0*.*0004*	*0*.*9775*±*0*.*0028*	*0*.*9876*±*0*.*0015*	*0*.*9399*±*0*.*0108*	*0*.*0225*±*0*.*0028*
*32×32 Mosaic*	*0*.*9993*±*0*.*0001*	*0*.*9946*±*0*.*0005*	*0*.*9969*±*0*.*0003*	*0*.*9890*±*0*.*0021*	*0*.*0054*±*0*.*0005*
***32×32 Mosaic centralized***	***0*.*9995*±*0*.*0001***	***0*.*9949*±*0*.*0004***	***0*.*9972*±*0*.*0002***	***0*.*9933*±*0*.*0006***	***0*.*0051*±*0*.*0004***

**Table 2:** 29-bit CAN ID test results for all four attacks.

***Threshold = 0*.*5***	*Prec*	*Rec*	*F1_score*	*Acc*	*FNR*
*29×29 sequential*	*0*.*9959*±*0*.*0003*	*0*.*9827*±*0*.*0026*	*0*.*9892*±*0*.*0013*	*0*.*9918*±*0*.*0010*	*0*.*0173*±*0*.*0026*
*48×48 Mosaic*	*0*.*9964*±*0*.*0005*	*0*.*9889*±*0*.*0015*	*0*.*9926*±*0*.*0007*	*0*.*9943*±*0*.*0005*	*0*.*0111*±*0*.*0015*
***48×48 Mosaic centralized***	***0*.*9979*±*0*.*0006***	***0*.*9896*±*0*.*0016***	***0*.*9937*±*0*.*0006***	***0*.*9952*±*0*.*0004***	***0*.*0104*±*0*.*0016***
***Threshold = 0*.*6***	*Prec*	*Rec*	*F1_score*	*Acc*	*FNR*
*29×29 sequential*	*0*.*9965*±*0*.*0003*	*0*.*9848*±*0*.*0023*	*0*.*9906*±*0*.*0011*	*0*.*9906*±*0*.*0011*	*0*.*0152*±*0*.*0023*
*48×48 Mosaic*	*0*.*9971*±*0*.*0005*	*0*.*9901*±*0*.*0013*	*0*.*9935*±*0*.*0006*	*0*.*9936*±*0*.*0006*	*0*.*0100*±*0*.*0013*
***48×48 Mosaic centralized***	***0*.*9984*±*0*.*0004***	***0*.*9907*±*0*.*0014***	***0*.*9945*±*0*.*0006***	***0*.*9945*±*0*.*0005***	***0*.*0093*±*0*.*0014***
***Threshold = 0*.*7***	*Prec*	*Rec*	*F1_score*	*Acc*	*FNR*
*29×29 sequential*	*0*.*9971*±*0*.*0003*	*0*.*9867*±*0*.*0020*	*0*.*9919*±*0*.*0009*	*0*.*9890*±*0*.*0013*	*0*.*0133*±*0*.*0020*
*48×48 Mosaic*	*0*.*9978*±*0*.*0004*	*0*.*9913*±*0*.*0012*	*0*.*9945*±*0*.*0006*	*0*.*9926*±*0*.*0007*	*0*.*0087*±*0*.*0012*
***48×48 Mosaic centralized***	***0*.*9988*±*0*.*0003***	***0*.*9919*±*0*.*0011***	***0*.*9953*±*0*.*0005***	***0*.*9935*±*0*.*0006***	***0*.*0081*±*0*.*0011***
***Threshold = 0*.*8***	*Prec*	*Rec*	*F1_score*	*Acc*	*FNR*
*29×29 sequential*	*0*.*9976*±*0*.*0003*	*0*.*9889*±*0*.*0016*	*0*.*9932*±*0*.*0007*	*0*.*9866*±*0*.*0015*	*0*.*0111*±*0*.*0016*
*48×48 Mosaic*	*0*.*9984*±*0*.*0002*	*0*.*9924*±*0*.*0009*	*0*.*9954*±*0*.*0004*	*0*.*9911*±*0*.*0008*	*0*.*0076*±*0*.*0009*
***48×48 Mosaic centralized***	***0*.*9991*±*0*.*0003***	***0*.*9931*±*0*.*0010***	***0*.*9961*±*0*.*0004***	***0*.*9922*±*0*.*0007***	***0*.*0069*±*0*.*0010***
***Threshold = 0*.*9***	*Prec*	*Rec*	*F1_score*	*Acc*	*FNR*
*29×29 sequential*	*0*.*9984*±*0*.*0002*	*0*.*9916*±*0*.*0012*	*0*.*9950*±*0*.*0005*	*0*.*9809*±*0*.*0024*	*0*.*0084*±*0*.*0012*
*48×48 Mosaic*	*0*.*9989*±*0*.*0001*	*0*.*9940*±*0*.*0008*	*0*.*9964*±*0*.*0004*	*0*.*9883*±*0*.*0011*	*0*.*0060*±*0*.*0008*
***48×48 Mosaic centralized***	***0*.*9994*±*0*.*0001***	***0*.*9945*±*0*.*0007***	***0*.*9969*±*0*.*0003***	***0*.*9894*±*0*.*0010***	***0*.*0055*±*0*.*0007***

When the 29-bit CAN ID was used, we first encoded the data to a 29×29 grid according to the direct coding method described in [30] as the input of the model. Then, the 29-bit CAN ID was first encoded into a 6×6 data grid and 8×8 of such data grids were put together to form a 48×48 Mosaic pattern as the input data of the model. Experimental results with the direct and the Mosaic coding methods and the centralized data processing method for the 29-bit CAN ID with all four attack data under different thresholds (0.5, 0.6, 0.7, 0.8 and 0.9) are shown in Table 2. We can see from Table 2 that the Mosaic coding methods also performed better in all evaluation indexes with generally lower standard deviation than that of 29×29 direct sequential coding method. We can also see that the centralized Mosaic coding method performed the best among all the evaluation indexes compared with other non-centralized methods.

**Fig 9:**
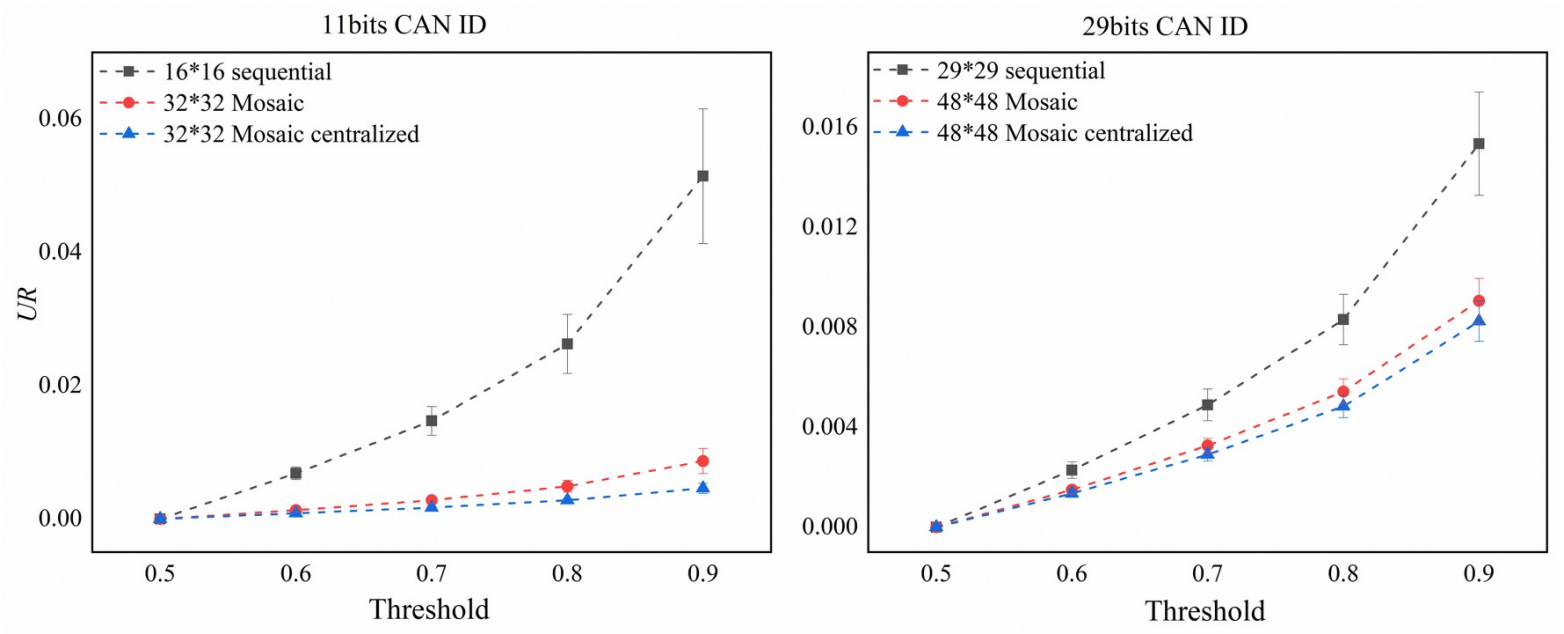
A comparison of *UR* values for the four attack types.

When the threshold is greater than 0.5, the larger the threshold is, the higher the model classification standard will be, and the larger the *UR* value will have. Under the same threshold condition, the smaller the *UR*, the better the reliability of the method, which means that more samples are concentrated around the two extreme values (0 or 1). The mean *UR* and the corresponding standard deviation of the 10 experiments of each coding method tested using the data set containing all four attack types are shown in Fig 9. It can be seen from this Figure that the *UR* value associated with the Mosaic coding method is significantly lower than that of the direct coding method under the same threshold for both 11-bit and 29-bit CAN ID data with much lower standard deviation, which shows that the Mosaic coding method used in this paper is more reliable and runs more stable. The *UR* value for the Mosaic coding after data centralization was further reduced compared to that without centralized Mosaic coding, indicating the effectiveness of data centralization.

## Discussions

In order to further illustrate the reliability of the method presented in this paper and to more truly simulate the possibility of different combinations of attacks in real attack situations, the model was also tested with different combinations of attacks in this paper. The CNN models previously trained on data sets containing all four attack types were first saved. Next, the remaining 25% of the randomly selected test set was combined to form 14 data sets with different combinations of attack types. Combinations of attacks considered in this paper can be divided into three categories: (1) three types of attacks; (2) two types of attacks; and (3) a single type of attack. The datasets were numbered sequentially as shown in Table 3.

**Table 3:** Data set combinations and their numbers.

Number	DataSet	Number	DataSet
***1***	*Normal_DoS_Fuzzy_Gear*	***8***	*Normal_Fuzzy_Gear*
***2***	*Normal_DoS_Fuzzy_RPM*	***9***	*Normal_Fuzzy_ RPM*
***3***	*Normal_DoS_Gear_RPM*	***10***	*Normal_Gear_ RPM*
***4***	*Normal_Fuzzy_Gear_RPM*	***11***	*Normal_DoS*
***5***	*Normal_DoS_Fuzzy*	***12***	*Normal_Fuzzy*
***6***	*Normal_DoS_Gear*	***13***	*Normal_Gear*
***7***	*Normal_DoS_RPM*	***14***	*Normal_RPM*

**Fig 10:**
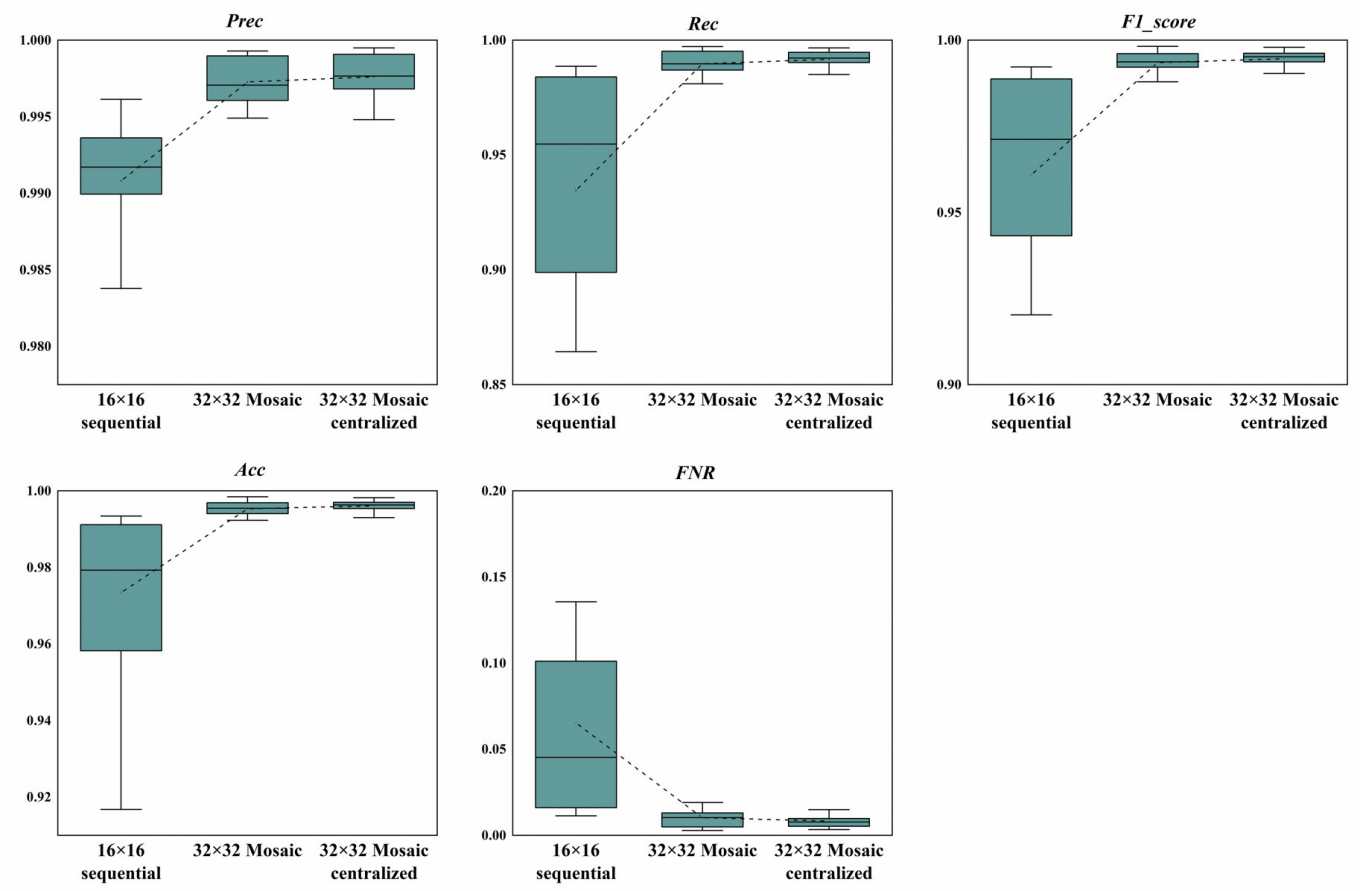
Comparison of the evaluation indexes with 11-bit CAN ID. The dotted lines are the mean value of each boxplot.

**Fig 11:**
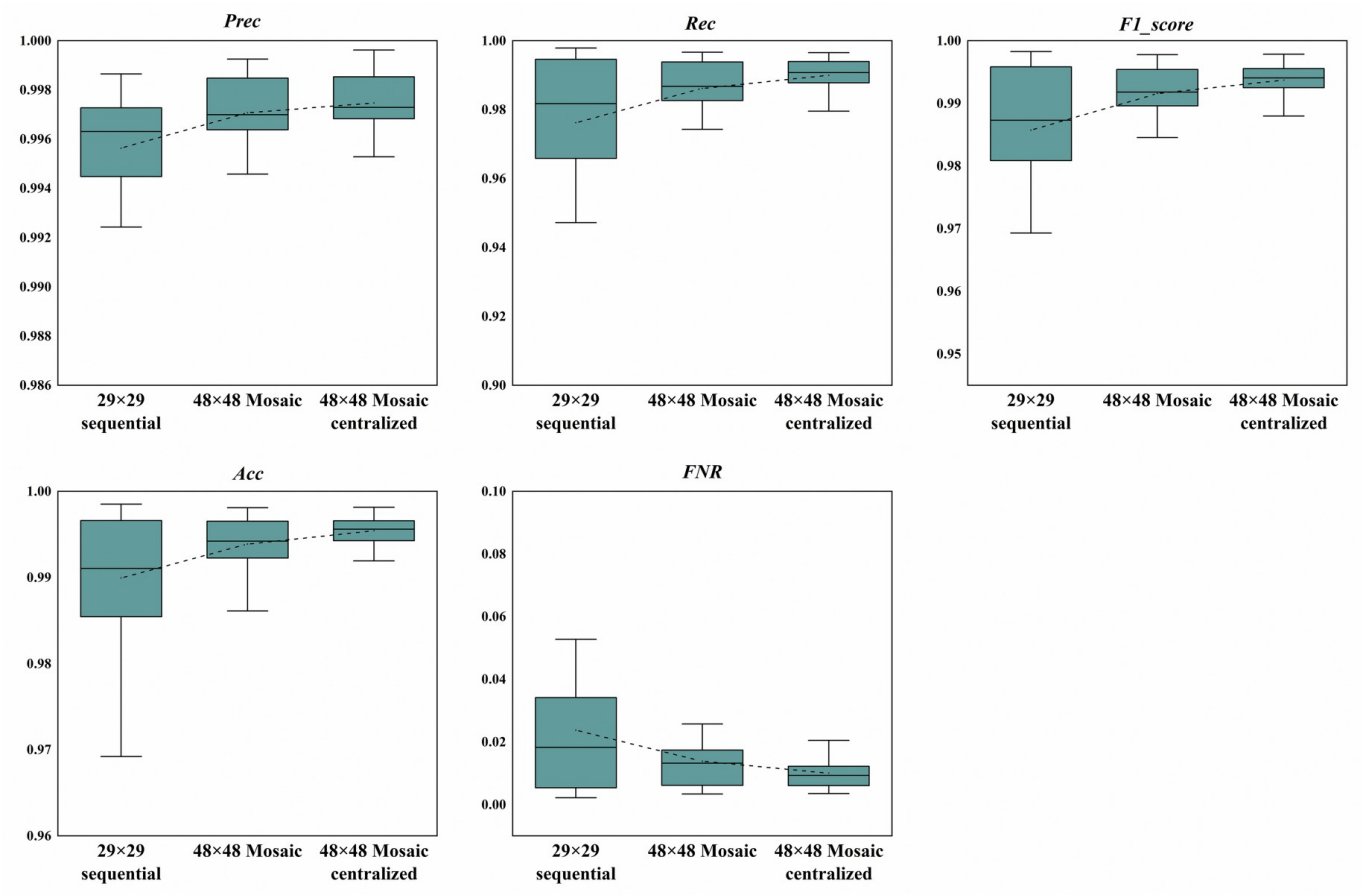
Comparison of the evaluation indexes with 29-bit CAN ID. The dotted lines are the mean value of each boxplot.

The above 14 combinations of different attacks were tested respectively using the models with the threshold value of 0.5, and the resultant evaluation indexes of each model in the 14 data sets were compared in the form of boxplot. The results of the 11-bit and 29-bit CAN ID tests are shown in Figs 10 and 11. The dotted lines on these figures are the mean value of each boxplot. We can see from these figures that the Mosaic coding method could not only achieve better results in all the indexes compared with the direct sequential coding method, it also showed a lower variation for different attack combinations. This means our model is more stable and reliable for different combinations of attacks with higher detecting accuracy, which is better in dealing with real-world attacks.

Analyzing the above boxplot and our specific result data, we also found that no matter whether the 11-bit or the 29-bit CAN IDs were used, the combinations of attacks numbered 5 (*Normal_DoS_Fuzzy*) and 12 (*Normal_Fuzzy*) performed the worst over all evaluation indexes under different coding methods. However, when the combination numbered 13 (*Normal_Gear*) was used, the overall performance was relatively good. Nevertheless, whenever the Fuzzy attack was not included in the test combination, the results achieved by direct sequential coding method were not so bad, but they could always be further improved by our Mosaic coding method. If the test combination contained Fuzzy attack, the direct sequential coding method performed relatively poor, but the Mosaic coding method could always make some obvious improvements. In short, the Mosaic and centralized Mosaic coding methods always performed better than the sequential coding method with much lower variation over different combinations of attacks.

**Fig 12:**
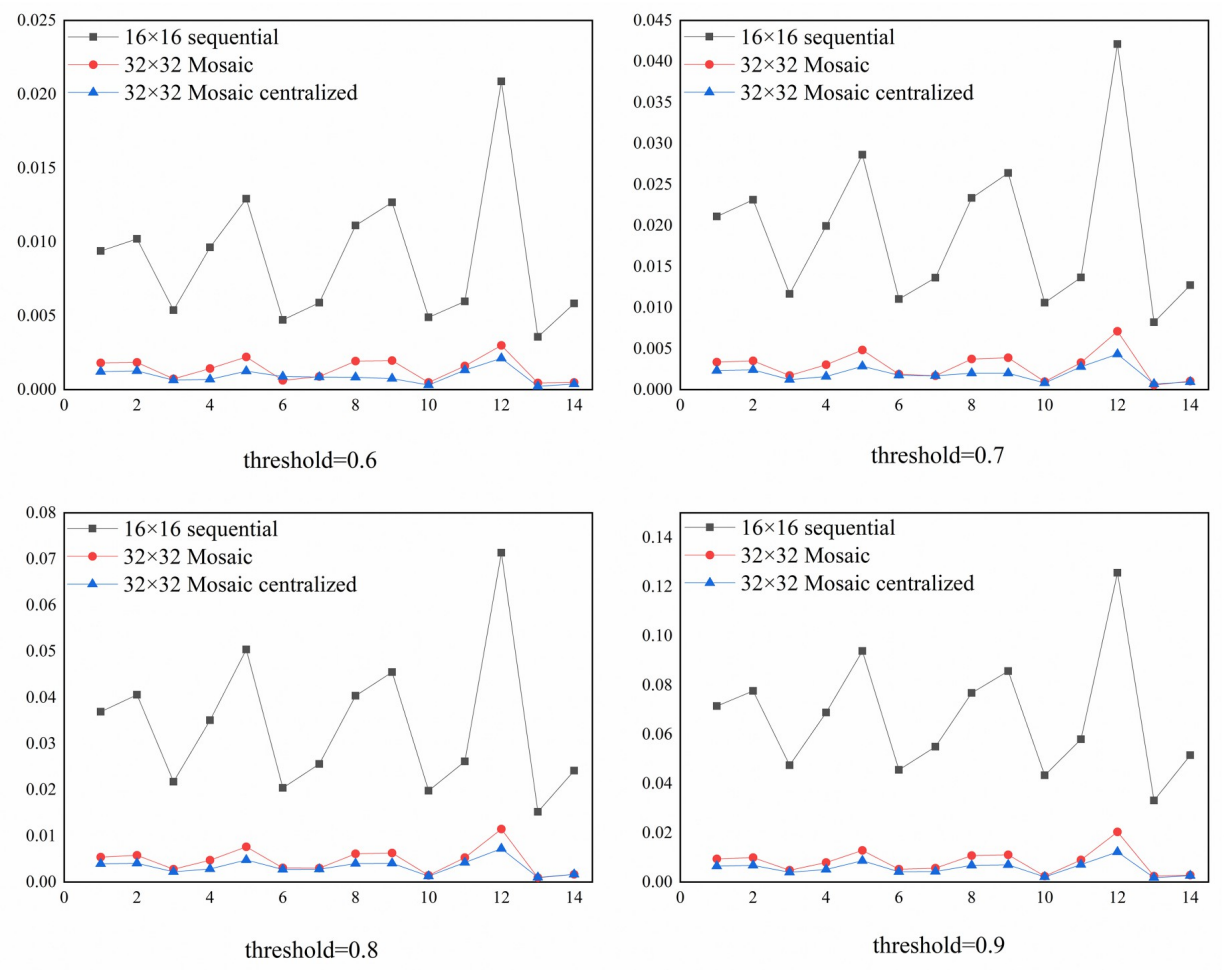
Comparison of *UR* for the 11-bit CAN ID under different thresholds.

**Fig 13:**
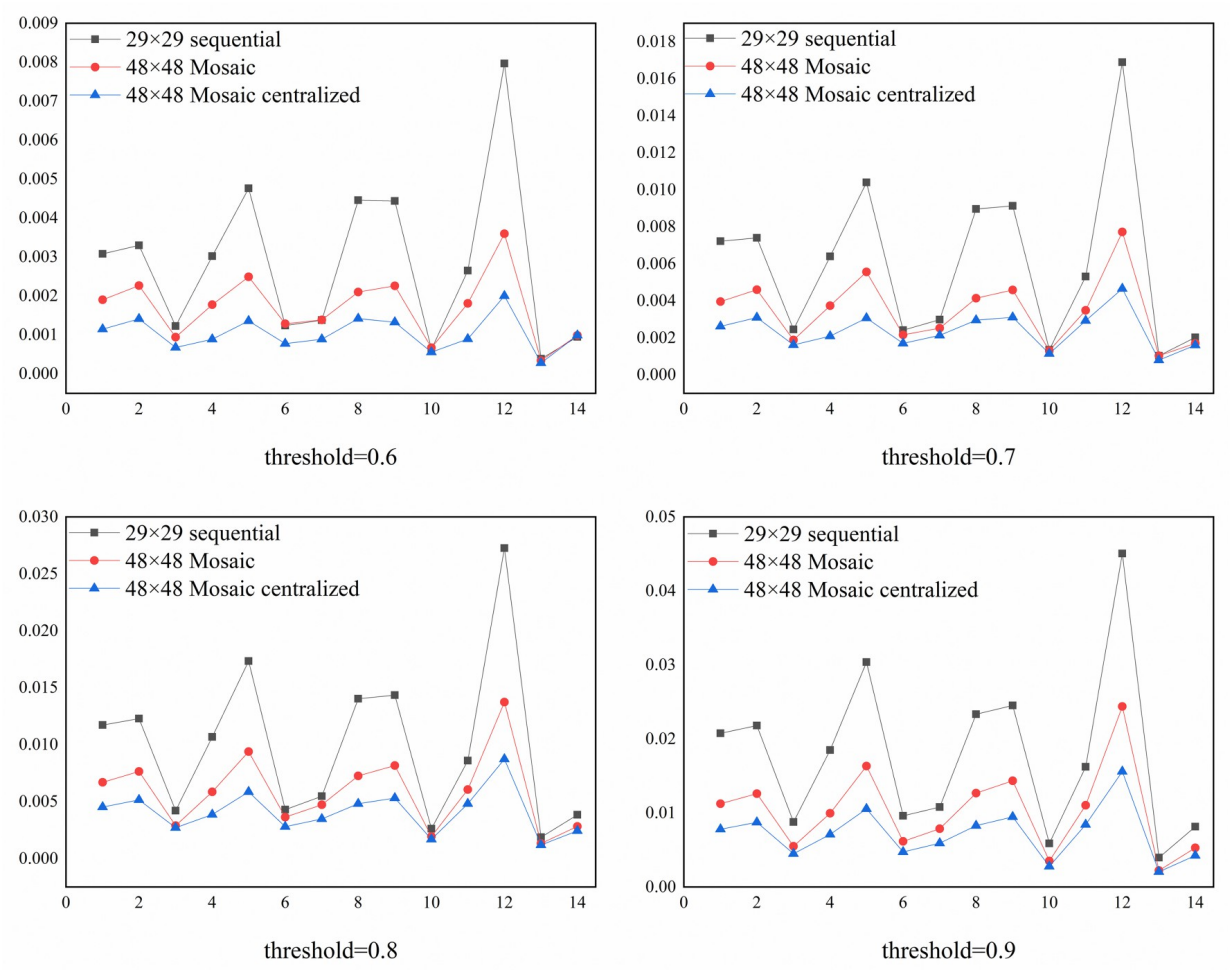
Comparison of *UR* for the 29-bit CAN ID under different thresholds.

When the threshold is greater than 0.5, some of the test samples will not be recognized by the network. Therefore, Figs 12 and 13 compared the *UR* of 11-bit and 29-bit CAN IDs respectively under different thresholds. In these figures, 14 different combinations of attack test data sets were tested and the numbers on the horizontal coordinates represent the indicator of the data set given in Table 3. We can see from these figures that, in both the 11-bit and 29-bit CAN IDs, the Mosaic coding method presented a lower *UR* value compared to the direct sequential coding method and centralized Mosaic coding further reduced the *UR* value. We also find that the Mosaic coding method could not only achieve better results in all the combinations compared with the direct sequential coding method, it also showed a lower variation for different attack combinations again. In addition, combinations 1, 2, 4, 5, 8, 9 and 12 are all associated with higher *UR*s, which all contain the Fuzzy attack. This showed that Fuzzy attack was the most difficult to be detected.

## Conclusion

An intrusion detection model based on the Mosaic and centralized Mosaic coded CNN was proposed in this paper to detect any combinations of data sets containing multiple CAN bus attack types to vehicles after being trained by only one full combination of the data set. Experimental results showed that, no matter whether the 11-bit or the 29-bit CAN ID was used, our model always performed better than the sequential coding method with much lower variations over the combinations with different numbers of attacks. The unrecognized rate was also the lowest with the lowest variation over different combinations of attacks when the threshold was greater than 0.5. Furthermore, the centralized method performed even better with higher stability over different combinations. This showed the feasibility of the method proposed in this paper in detecting different kinds of real attacks. In addition, since the real 29-bit CAN ID data set was also used in this paper, it also fits the future evolutions of the CAN bus to suit for more complex networks.
